# ECDC’s latest publications

**DOI:** 10.2807/1560-7917.ES.2019.24.50.1912122

**Published:** 2019-12-12

**Authors:** Eurosurveillance editorial team

**Affiliations:** 1 European Centre for Disease Prevention and Control (ECDC), Stockholm, Sweden

**Keywords:** infectious diseases, public health, Europe

 

**Rapid risk and outbreak assessments**

 

Cases of Lassa fever in the Netherlands ex Sierra Leone

Date of publication: 29 November 2019

https://www.ecdc.europa.eu/en/publications-data/rapid-risk-assessment-cases-lassa-fever-netherlands-ex-sierra-leone

**

Multi-country outbreak of *Listeria monocytogenes* sequence type 6 infections linked to ready-to-eat meat products

Date of publication: 26 November 2019

https://www.ecdc.europa.eu/en/publications-data/rapid-outbreak-assessment-multi-country-outbreak-listeria-monocytogenes-sequence

 

Sexual transmission of dengue in Spain

Date of publication: 18 November 2019

https://www.ecdc.europa.eu/en/publications-data/rapid-risk-assessment-sexual-transmission-dengue-spain

 

Outbreak of carbapenemase-producing and colistin-resistant *Klebsiella pneumoniae*, north-east Germany, 2019

Date of publication: 28 October 2019

https://www.ecdc.europa.eu/en/publications-data/outbreak-Klebsiella-pneumoniae-Germany

 

Zika virus disease in Var department, France

Date of publication: 16 October 2019

https://www.ecdc.europa.eu/en/publications-data/rapid-risk-assessment-zika-virus-disease-var-department-france

 

Autochthonous cases of dengue in Spain and France

Date of publication: 1 October 2019

https://www.ecdc.europa.eu/en/publications-data/rapid-risk-assessment-autochthonous-cases-dengue-spain-and-france

 

**Technical reports**

 

ECDC Evidence Brief: Pre-exposure prophylaxis for HIV prevention in Europe and Central Asia

Date of publication: 28 November 2019

https://www.ecdc.europa.eu/en/publications-data/ecdc-evidence-brief-pre-exposure-prophylaxis-hiv-prevention-europe-and-central

 

HIV testing, monitoring implementation of the Dublin Declaration on partnership to fight HIV/AIDS in Europe and Central Asia: 2018 progress report

Date of publication: 27 November 2019

https://www.ecdc.europa.eu/en/publications-data/hiv-testing-monitoring-implementation-dublin-declaration-partnership-fight

 

External quality assessment of laboratory performance – European Antimicrobial Resistance Surveillance Network (EARS-Net), 2018

Date of publication: 22 November 2019

https://www.ecdc.europa.eu/en/publications-data/external-quality-assessment-laboratory-performance-european-antimicrobial-0

 

Community and institutional public health emergency preparedness synergies - enablers and barriers

Date of publication: 19 November 2019

https://www.ecdc.europa.eu/en/publications-data/community-and-institutional-public-health-emergency-preparedness-synergies

 

Community engagement and institutional collaboration during outbreaks of Shiga toxin/verocytotoxin-producing *Escherichia coli* in Ireland

Date of publication: 19 November 2019

https://www.ecdc.europa.eu/en/publications-data/community-engagement-and-institutional-collaboration-during-outbreaks-shiga

 

Community engagement and institutional collaboration in Iceland during a norovirus outbreak at Úlfsljótsvatn Outdoor and Scout Centre (10–15 August 2017)

Date of publication: 19 November 2019

https://www.ecdc.europa.eu/en/publications-data/community-engagement-and-institutional-collaboration-iceland-during-norovirus

 

Survey of healthcare workers’ knowledge, attitudes and behaviours on antibiotics, antibiotic use and antibiotic resistance in the EU/EEA

Date of publication: 18 November 2019

https://www.ecdc.europa.eu/en/publications-data/survey-healthcare-workers-knowledge-attitudes-and-behaviours-antibiotics

 

A spatial modelling method for vector surveillance

Date of publication: 14 November 2019

https://www.ecdc.europa.eu/en/publications-data/spatial-modelling-method-vector-surveillance

 

Health emergency preparedness for imported cases of high-consequence infectious diseases

Date of publication: 22 October 2019

https://www.ecdc.europa.eu/en/publications-data/health-emergency-preparedness-imported-cases-high-consequence-infectious-diseases

 

Managing heterogeneity when pooling data from different surveillance systems

Date of publication: 21 October 2019

https://www.ecdc.europa.eu/en/publications-data/managing-heterogeneity-when-pooling-data-different-surveillance-systems

 

HIV and people who inject drugs - Monitoring implementation of the Dublin Declaration

Date of publication: 9 October 2019

https://www.ecdc.europa.eu/en/publications-data/hiv-and-people-who-inject-drugs-monitoring-implementation-dublin-declaration

 

Response plan to control and manage the threat of multi- and extensively drug-resistant gonorrhoea in Europe

Date of publication: 7 October 2019

https://www.ecdc.europa.eu/en/publications-data/response-plan-control-and-manage-threat-multi-and-extensively-drug-resistant

 

**Surveillance reports**

 

HIV/AIDS surveillance in Europe 2019 - 2018 data

Date of publication: 28 November 2019

https://www.ecdc.europa.eu/en/publications-data/hivaids-surveillance-europe-2019-2018-data

 

Surveillance of antimicrobial resistance in Europe 2018

Date of publication: 11 November 2019

https://www.ecdc.europa.eu/en/publications-data/surveillance-antimicrobial-resistance-europe-2018

 

Influenza virus characterisation, Summary Europe, October 2019

Date of publication: 11 November 2019

https://www.ecdc.europa.eu/en/publications-data/influenza-virus-characterisation-october-2019

 

Monthly measles and rubella monitoring report, November 2019

Date of publication: 11 November 2019

https://www.ecdc.europa.eu/en/publications-data/monthly-measles-and-rubella-monitoring-report-november-2019

 

Influenza virus characterisation, Summary Europe, September 2019

Date of publication: 14 October 2019

https://www.ecdc.europa.eu/en/publications-data/influenza-virus-characterisation-september-2019

 

Monthly measles and rubella monitoring report, October 2019

Date of publication: 11 October 2019

https://www.ecdc.europa.eu/en/publications-data/monthly-measles-and-rubella-monitoring-report-october-2019

 

**Annual Epidemiological Report series on communicable diseases in Europe**

****

**Published in November:**

****

Chapter on antimicrobial consumption for 2018

https://www.ecdc.europa.eu/en/publications-data/surveillance-antimicrobial-consumption-europe-2018

 

Chapter on toxoplasmosis for 2017

https://www.ecdc.europa.eu/en/publications-data/congenital-toxoplasmosis-annual-epidemiological-report-2017

 

Chapter on rabies for 2018

https://www.ecdc.europa.eu/en/publications-data/rabies-annual-epidemiological-report-2018

 

Chapter on smallpox for 2018

https://www.ecdc.europa.eu/en/publications-data/smallpox-annual-epidemiological-report-2018

 

Chapter on yersiniosis for 2018

https://www.ecdc.europa.eu/en/publications-data/yersiniosis-annual-epidemiological-report-2018

 

**Published in October:**

****

Chapter on cryptosporidiosis for 2017

https://www.ecdc.europa.eu/en/publications-data/cryptosporidiosis-annual-epidemiological-report-2017

 

Chapter on healthcare-associated infections in intensive care units for 2017

https://www.ecdc.europa.eu/en/publications-data/healthcare-associated-infections-intensive-care-units-annual-epidemiological-1

 

Chapter on healthcare-associated infections: surgical site infections for 2017

https://www.ecdc.europa.eu/en/publications-data/healthcare-associated-infections-surgical-site-infections-annual-1

 

**ECDC communicable disease threats report**

Date of publication: every Friday

https://ecdc.europa.eu/en/threats-and-outbreaks/reports-and-data/weekly-threats

**

**Scientific peer-reviewed publications**

Publications by ECDC staff in scientific journals

Updated monthly

https://ecdc.europa.eu/en/publications-data?f%5b0%5d=output_types%3A1247

